# Synergistic Effects of Weightlessness, Isoproterenol, and Radiation on DNA Damage Response and Cytokine Production in Immune Cells

**DOI:** 10.3390/ijms19113689

**Published:** 2018-11-21

**Authors:** Maria Moreno-Villanueva, Alan H. Feiveson, Stephanie Krieger, AnneMarie Kay Brinda, Gudrun von Scheven, Alexander Bürkle, Brian Crucian, Honglu Wu

**Affiliations:** 1 National Aeronautics and Space Administration (NASA), Johnson Space Center Houston, Houston, TX 77058, USA; alan.h.feiveson@nasa.gov (A.H.F.); brian.crucian-1@nasa.gov (B.C.); honglu.wu-1@nasa.gov (H.W.); 2 Molecular Toxicology Group, Department of Biology, Box 628, University of Konstanz, 78457 Konstanz, Germany; gudrun.vonscheven@uni-konstanz.de (G.v.S.); alexander.buerkle@uni-konstanz.de (A.B.); 3 Department of Biomedical Engineering, University of Minnesota, 312 Church Street SE, Minneapolis, MN 55455, USA; kovac149@umn.edu; 4 KBRwyle, 2400 NASA Parkway, Houston, TX 77058, USA; stephanie.s.krieger@nasa.gov

**Keywords:** radiation, simulated microgravity, DNA damage response, adrenergic receptor, cytokines

## Abstract

The implementation of rotating-wall vessels (RWVs) for studying the effect of lack of gravity has attracted attention, especially in the fields of stem cells, tissue regeneration, and cancer research. Immune cells incubated in RWVs exhibit several features of immunosuppression including impaired leukocyte proliferation, cytokine responses, and antibody production. Interestingly, stress hormones influence cellular immune pathways affected by microgravity, such as cell proliferation, apoptosis, DNA repair, and T cell activation. These pathways are crucial defense mechanisms that protect the cell from toxins, pathogens, and radiation. Despite the importance of the adrenergic receptor in regulating the immune system, the effect of microgravity on the adrenergic system has been poorly studied. Thus, we elected to investigate the synergistic effects of isoproterenol (a sympathomimetic drug), radiation, and microgravity in nonstimulated immune cells. Peripheral blood mononuclear cells were treated with the sympathomimetic drug isoproterenol, exposed to 0.8 or 2 Gy γ-radiation, and incubated in RWVs. Mixed model regression analyses showed significant synergistic effects on the expression of the β2-adrenergic receptor gene (ADRB2). Radiation alone increased ADRB2 expression, and cells incubated in microgravity had more DNA strand breaks than cells incubated in normal gravity. We observed radiation-induced cytokine production only in microgravity. Prior treatment with isoproterenol clearly prevents most of the microgravity-mediated effects. RWVs may be a useful tool to provide insight into novel regulatory pathways, providing benefit not only to astronauts but also to patients suffering from immune disorders or undergoing radiotherapy.

## 1. Introduction

The rotating-wall vessel (RWV) bioreactor is a suspension culture system that provides the necessary oxygen and nutrients for cells to develop and polarize while they grow under low fluid-shear conditions. The low fluid-shear environment also promotes the colocalization of particles (e.g., cells and/or beads) that allows cells to interact and form aggregates [1]. National Aeronautics and Space Administration (NASA) engineers developed the RWV bioreactor system to model space-like microgravity conditions [2,3,4], however researchers are also using this technology in the fields of cancer [5], tissue engineering [6,7], and regenerative medicine [8,9]. When cells are exposed to simulated microgravity several biological processes become dysregulated, including T cell regulation, apoptosis, DNA repair, and cell proliferation, differentiation, and migration [10,11,12,13,14]. Although many investigators have studied the effects of microgravity and radiation on genes and proteins involved in DNA damage response (DDR), the mechanisms are not well understood. During stress situations, endogenous release of stress hormones affects cellular responses to exogenous factors such us microgravity, radiation, and pathogens. Therefore, we investigated how activated adrenergic receptors and microgravity affect DNA damage response and cytokine production in irradiated cells. 

### 1.1. Exogenous Factors Affecting DNA Damage Response (DDR)

DDR includes mechanisms for detection, processing, and repair of DNA lesions [15]. Thus, impaired DDR leads to the accumulation of DNA lesions, which increase the risk of genomic instability. Simulated microgravity was found to decrease the expression of DNA repair genes involved in mismatch repair (MMR), base excision repair (BER), and nucleotide excision repair (NER), resulting in the accumulation of DNA damage [16] and an increase in PARP-1 activity [17]. DNA damage can signal repair processes, but it can also trigger apoptosis [18], a secondary response that removes damaged cells to protect tissue function [19]. Immune cells undergo apoptosis during pathological conditions (excessive DNA damage) or physiological processes (deletion of immune cells recognizing self-antigens). In both cases, dysregulation of apoptotic pathways might be detrimental to the integrity of the immune system and could lead to autoimmune disease and immunodeficiency. Genes involved in cell proliferation (CyclinD1 and PCNA) and apoptosis (BAX are downregulated in lymphocytes that are grown in simulated microgravity [16]. Moreover, simulated microgravity has been shown to inhibit radiation-induced apoptosis and activation-induced cell death in stimulated lymphocytes [20]. In stimulated primary CD4+ T cells, p21 mRNA levels increased 4.1-fold after 20s in microgravity during parabolic flight compared to levels in 1*g* controls [21]. These results suggest that T lymphocyte proliferation requires Earth gravity and that the increased expression of cell cycle regulatory proteins contributes to immune depression in space [21]. 

In general, radiation induces apoptosis but the specific response depends on the radiation dose. For example, when mouse splenocytes were exposed to 5 doses of γ-radiation ranging from 0.01 to 2 Gy, the low doses decreased apoptosis prominently in natural killer (NK) cells and dendritic cells (DCs) whereas 2 Gy increased apoptosis in all splenocyte subpopulations; B cells were the most sensitive to radiation whereas NK cells and DCs were the least sensitive [22]. Recent studies suggest that a combination of microgravity and low-dose radiation may decrease apoptosis but may potentially increase oxidative stress [23]. Furthermore, a decreased apoptosis rate has been observed in fetal fibroblasts 24 h after exposure to either moderate (0.5 and 1 Gy) or high (4 Gy) doses of X-rays under simulated microgravity [24]. Lymphoblastoid TK6 cells irradiated with γ-rays and incubated for 24 h in a simulated microgravity environment showed significantly less apoptosis, an increased number of cells in G1 cell cycle phase, and a higher frequency of mutations and micronucleated cells than cells maintained in 1*g* [25]. These results suggest that a combination of microgravity and radiation (at least γ-rays) reduces the rate of apoptosis induced with radiation alone, and, therefore, microgravity increases the frequency of damaged cells that survive after irradiation.

### 1.2. Endogenous Factors Affecting DNA Damage Response

Both exogenous factors, such as radiation or absence of gravity, and endogenous factors, such as release of stress hormones or the presence of inflammatory processes, might affect, either directly or indirectly, the integrity of DNA in immune cells, thereby compromising immune function. Lymphocytes are exposed to genotoxic stresses during immune responses (accidental DNA damage) and during development and maturation (programmed DNA damage). Immune cells also incur DNA damage during infectious and inflammatory processes and this triggers the activation of DNA repair pathways. Interestingly, Fontes and colleagues reported recently that DNA repair can affect host immune responses and inflammation [26].

Furthermore, exposure to stress affects the immune system’s ability to produce antibodies, making organisms more vulnerable to infections [27]. An immune dysfunction under stress can be due to imbalances in the release of stress hormones, which subsequently activate the receptor-mediated signal. There is considerable evidence that adrenergic pathways are involved in immune system regulation. Although adrenergic modulation of immune cells has been investigated [28], the mechanisms that convert psychological stress into cellular dysfunction are still poorly understood. Researchers have shown that exposure to stress activates NF-B, which coincides with a rapid increase in levels of catecholamines and cortisol in humans [29]. Adrenalin and noradrenalin bind to β-adrenergic receptors leading to an increase in intracellular cAMP, a second messenger involved in the activation of protein kinase A (PKA). In immune cells, cAMP acts as signal transducer in several physiological and pathological responses [30]. Both, cAMP and PKA have been associated with apoptosis. In the immune system, activation of cAMP signaling increases apoptosis in human B-precursor cells [31] and delays apoptosis in human neutrophils [32]. Furthermore, stimulation of the β-adrenergic receptor or addition of exogenous cAMP can induce apoptosis in thymocytes [33]. Interestingly, activation of cAMP signaling inhibits DNA radiation-induced apoptosis in B cell precursor acute lymphoblastic leukemia (BCP-ALL) [34]. Moreover, cAMP plays an important role in several immunological processes such as cytolytic activity, antibody production, and cell proliferation. Activated adrenergic receptors trigger the cAMP response element binding protein (CREB), inducing the transcription of genes encoding for a variety of cytokines [35]. The adenylyl cyclase-cAMP system inhibits IFN-gamma, TNF-alpha-stimulated production of T Cell-Directed CC Chemokine (TARC), and Chemokine (C-C Motif) Ligand 22 (CCL22) through the NF-B and MAPK pathways [36]. Furthermore, macrophages and lymphocytes provide endogenous sources of catecholamines that regulate inflammatory responses [37]. 

The effect of microgravity on adrenergic pathways is not fully understood. Early studies reported an increase in epinephrine-induced responsiveness in human subjects during head-down bed rest to simulate microgravity [38,39]. More recent studies have shown altered levels of salivary proteins that are associated with β-adrenergic signaling, including a study of cAMP and PKA in mice flown in space [40]. Also, simulated microgravity activates the β-adrenergic receptor leading to the formation of cAMP and activation of PKA and cAMP response element binding protein (CREB) in human neural stem cells (hNSCs) [41]. Neuroendocrine hormones such as cortisol and catecholamines are altered in astronauts during spaceflight [42,43], and levels of norepinephrine and epinephrine in the astronauts’ plasma increase after spaceflight [44]. Adrenergic receptors are present in many cell types including immune cells. The sympathetic nervous system regulates and modulates cell differentiation and function during the immune response. For example, norepinephrine-activated β_2_-AR increases gene expression of inflammatory cytokines and reduces cell proliferation related cytokines [45]. Adrenaline and cortisone suppress CD69 expression in natural killer cells and this affects the early stages of proliferation and differentiation of these cells [46]. T helper cells (CD4) are the most prolific cytokine producers. This subset of cells can be subdivided into Th1 and Th2, which produce cytokines known as type-1 and type-2 cytokines, respectively [47]. The density of β_2_-AR in peripheral blood mononuclear cells (PBMCs) helps mediate the differential catecholamine effects on cytokine production favoring Th2 responses [48]. Consequently, β-adrenergic agonists shift the balance of human type-1/type-2 cytokine toward a type-2 response [49]. A Th2 response counteracts the Th1-mediated proinflammatory responses responsible for killing intracellular parasites [47]. Therefore, impaired β_2_-AR signaling due to radiation, microgravity, or the combination of both may also affect immune response. Crucian and colleagues monitored the concentrations of plasma cytokines in 28 astronauts during long-duration spaceflight onboard the International Space Station (ISS) and found increased levels of the following: Tumor necrosis factor-α (TNFα), IL-8, IL-1 ra, thrombopoietin (Tpo), vascular endothelial growth factor (VEGF), C-C motif chemokine ligand 2 (CCL2), chemokine ligand 4/macrophage inhibitory protein 1b (CCL4), and C-X-C motif chemokine 5/epithelial neutrophil-activating protein 78 (CXCL5) [50]. Cytokine response to radiation varies. For instance, expression of T-helper 1 (Th1) and T-helper 2 (Th2)-type cytokines decreased after low doses of radiation and increased after high doses; IL-6 reacted at early times and IL-10 at later times after radiation exposure; and IL-5 levels were higher in mice after total-body irradiation [22]. 

In the present study, we assessed whether activation of adrenergic response affects or changes how immune cells respond to radiation in simulated microgravity. We measured apoptosis, residual DNA damage and the expression of genes involved in DNA damage response, as well as cytokine production as a marker of immune cell activation.

## 2. Results

### 2.1. Optimization of the Cell Concentration and Rotation Speed in Rotatory Wall Vessels (RWVs)

**Figure 1:**
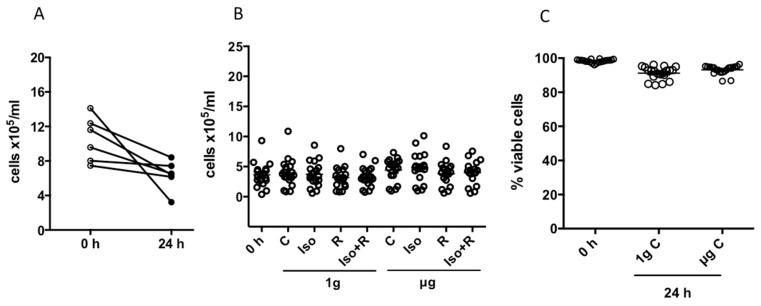
Cell recovery and cell viability after 24 h incubation in rotating-wall vessels (RWVs): (**A**) Change in the initial cell concentration with incubation time at a rotation speed of 10 rpm (6 independent experiments); (**B**) cell recovery of nontreated and treated cells over experimental conditions before and after 24 h incubation in RWVs rotating at 8.5 rpm (18 independent experiments); and (**C**) the percentage of live nontreated cells that remained alive after 24 h incubation rotating at a speed of 8.5 rpm (20 independent experiments).

Hammond et al. [51] summarized the principles for determining the optimal conditions for suspension cultures. Rotation of RVW’s is important for eliminating the gravity vector, but it is also important to minimize mechanical stress due to excessive speed. The manufacturer’s operation manual advises that cells not requiring microcarrier beads or scaffolding should be cultured at a minimum of 200,000 cells/mL, and lymphocytes should be rotated at 8 to 10 rpm [52]. Accordingly, to ensure that our experimental culture conditions did not lead to cell loss or excessive cell death rates, we determined the optimal cell concentration and rotation speed empirically. Fresh isolated PBMCs were treated with isoproterenol, radiation, or both and incubated in RWVs for 24 h as indicated in the methods section. When we introduced 8–12 × 10^5^ cells/mL into the vessel and rotated them at 10 rpm, we recovered a reduced number of cells after 24 h (Figure 1A). However, when we introduced 3.5–4.5 × 10^5^ cells/mL into the vessel and rotated them at 8.5 rpm for 24 h, we recovered approximately the same number of cells for all experimental conditions (Figure 1B). In addition, when the cells were rotated at 8.5 rpm, the average fraction of living cells decreased only slightly after 24 h (from 98.3% to 91.3% for 1*g* and to 93.2% for µg), indicating an acceptable level of cell death due to the ex vivo culture conditions (Figure 1C). 

### 2.2. Synergistic Effect of Gravity, Radiation and Isoproterenol on Apoptosis

**Figure 2:**
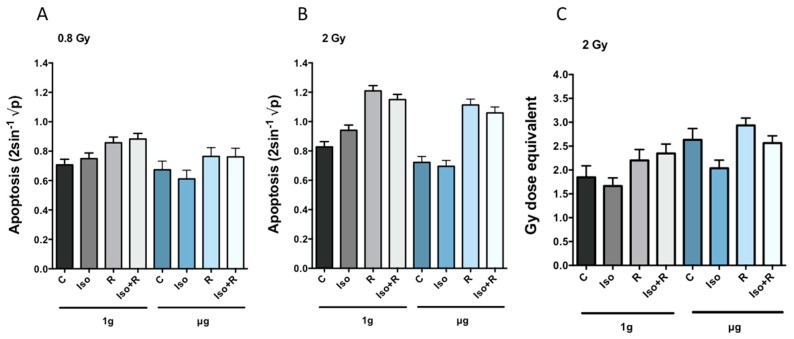
Apoptosis rate (calculated as indicated in Table 1) and residual DNA strand breaks after 24 h incubation in RWVs in µg or 1*g*. Apoptosis rate is represented by the mean transformed values (**A**) and (**B**) and DNA strand breaks are means expressed in Gy dose equivalent (**C**). Nontreated cells (C), isoproterenol (Iso) and radiation (R) treated cells. Error bars represent +1 SEM. Statistical analysis are summarized in Table 1.

A radiation dose of 0.8 Gy significantly increased the apoptosis rate in cells in 1*g*, whereas the rate of apoptosis in cells in µg after 0.8 Gy was not significantly different from nonradiated cells (Figure 2A). The number of apoptotic cells increased significantly in both gravity conditions after exposure to 2 Gy of radiation (Figure 2B). Treatment with isoproterenol before 2 Gy irradiation slightly but significantly reduced apoptosis rate (Iso × R (1*g*) interaction. This synergistic effect between isoproterenol and radiation was only observed in 1*g*. In µg there was far less evidence of this synergy (Iso × R (µg) interaction (Table 1). When cells undergo apoptosis, endogenous endonucleases cleave nuclear DNA into chromosomal fragments inducing DNA strand breaks [53]. Thus, we also measured accumulated DNA strand breaks. As expected, an increased radiation-induced apoptosis rate was accompanied by an increase in the amount of residual DNA strand breaks. Furthermore, in agreement with previous findings [54], the number of DNA strand breaks induced by microgravity in non-irradiated cells was higher than in cells incubated in 1*g*. This microgravity-associated accumulation of DNA strand breaks was prevented by previous treatment with isoproterenol (Figure 2C). 

### 2.3. Effects of Radiation, Microgravity, and Isoproterenol on Gene Expression

**Figure 3:**
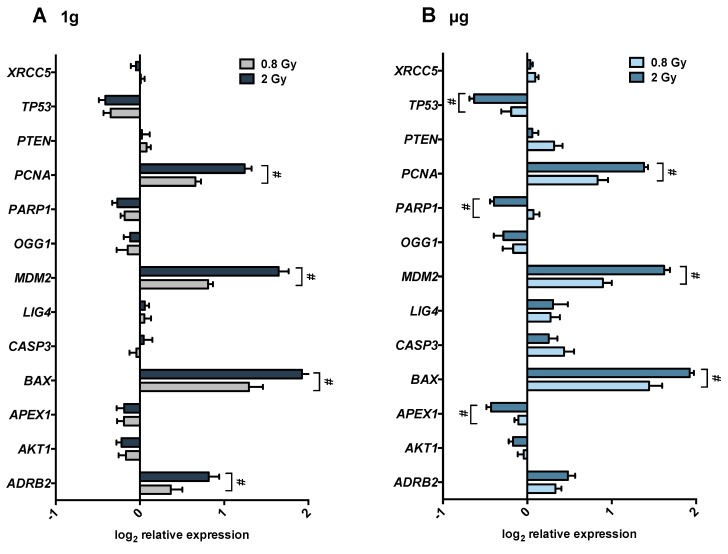
Radiation effects on gene expression relative to non-irradiated cells incubated in (**A)** 1*g* and (**B**) µg. Cells were irradiated either with 0.8 Gy (light bars) or 2 Gy (dark bars) and immediately incubated in 1*g* (grey bars) or µg (blue bars) for 24 h. Error bars represent SEM. Asterisks represent significant differences in gene expression in cells irradiated with 0.8 Gy compared to cells irradiated with 2 Gy. Statistical method: Krieger. *p*-value threshold: 0.018 after controlling the FDR (false-discovery rate—see statistical methods) to 5%.

The effects of 0.8 Gy and 2 Gy of radiation on gene expression are shown in Figure 3. In normal gravity (Figure 3A), the genes, BAX, MDM2, and PCNA were upregulated in response to both doses of radiation, but were significantly more upregulated after the dose of 2 Gy. We observed a similar pattern of expression for these genes in cells incubated in µg (Figure 3B). ADRB2 expression induced by 2 Gy irradiation was significantly greater in 1*g* than in µg. TP53, PARP1, and APEX1 were significantly more downregulated after 2 Gy than 0.8 Gy in cells incubated in µg, whereas no dose response was detected in cells incubated 1 g.

For the cells irradiated with 0.8 Gy or 2 Gy, all effects for each of 15 genes (a total of 180 contrasts) were estimated and tested for significance. A synergistic effect between gravity and radiation, manifested in the R × *g* interaction, was statistically significant only for *LIG4* after 0.8 Gy and *ADRB2* after 2 Gy irradiation.

In the absence of radiation and isoproterenol, no significant overall changes in gene expression were observed between 1*g* and µg except for *PARP1*, which is downregulated in µg (Table 2). Interestingly, treatment with isoproterenol prior to radiation (Iso × R) appeared to prevent radiation-induced expression of *LIG4* and *XRCC5* in 1*g* as well as *CASP3*, *PTEN*, and *XRCC5* in µg for cells irradiated with 0.8 Gy. In cells irradiated with 2 Gy isoproterenol prevented radiation-induced *XRCC5* gene expression in µg. Finally, we also identified a three-way synergistic effect of isoproterenol, radiation, and gravity (noted as “Iso × R × *g*”) for *ADRB2* expression (Table 3) in cells irradiated with 2 Gy. While in 1*g*, isoproterenol prevents the 2 Gy radiation effect (negative Iso × *g* interaction), in µg previous treatment with isoproterenol increases the 2 Gy radiation effect (a positive Iso × *g* interaction). The difference (three-way interaction) illustrates that for this gene, the synergy between isoproterenol and radiation changes dramatically with the change in gravity.

### 2.4. Synergistic Effects of Gravity, Radiation, and Isoproterenol on Cytokine Release

**Figure 4:**
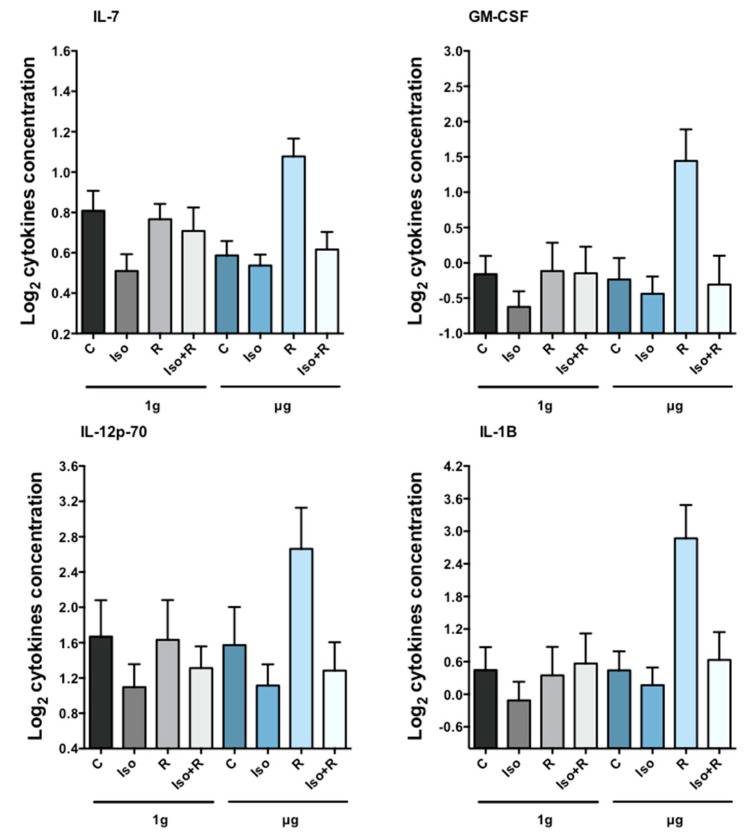
Cytokine concentration in cell culture medium. Cells were irradiated 2 Gy and subsequently incubated in 1g or µg. After 24 h cytokine concentration was measured. Radiation induced cytokine production in µg but not in 1*g*. Isoproterenol treatment prior to radiation prevented the production of all cytokines. Bars represent mean + 1 SEM from 10 independent experiments. The synergistic effect of isoproterenol and radiation in µg (Iso × R(µg)) was significant for all four cytokines. Statistical analyses are summarized in Table 4 and Table 5.

We estimated the contrast effects for 12 cytokines and tested for significant differences (N.B. Concentrations of IFNγ were often below detection limits and not analyzed). As with gene-expression and apoptosis results, we accounted for multiple testing (in this case 144 tests) by control of the FDR. For the 0.8 Gy irradiated samples, we found 18 significant contrasts after controlling the FDR to 5% (*p*-value threshold of 0.0068, Table 4). For the 2 Gy irradiated samples, control of the FDR to 1% (*p*-value threshold of 0.0049) resulted in 49 significant findings (Table 5). Radiation-induced cytokines were only detectable in cells incubated in simulated microgravity, and the concentration of cytokines was higher after irradiation with 2 Gy than 0.8 Gy (Figure 4A,B). This synergistic effect of radiation and gravity (R × *g*) was significant for GM-CSF, IL-2, IL-7, and TNFα in 2 Gy irradiated cells. The synergistic effect of isoproterenol and radiation in µg (Iso × R(µg)) was significant for GM-CSF, IL-12p70, IL-1B, and IL-7 in cells irradiated with 2 Gy and the synergistic effect of isoproterenol, radiation, and gravity (Iso × R × *g*) was significant for GM-CSF, IL-5, and IL-7 in cells irradiated with 2 Gy. 

## 3. Discussion

It has been reported that activated human T cells cultured in RWVs present alterations in expression of genes involved in several cellular processes such as signal transduction, DNA repair, apoptosis, immune and inflammatory responses, and metabolic pathways [55]. Decreased expression of genes involved in mismatch repair (MMR), base excision repair (BER), nucleotide excision repair (NER) as well as downregulation of p53 were observed in lymphocytes grown in simulated microgravity [16]. Our results are apparently contradictory to these findings, but it should be noted that the above-mentioned studies were conducted in proliferating lymphocytes. However, most of the time immune cells are in a quiescent, nonproliferating state. Nonetheless, our previous knowledge on DNA repair mechanisms has been gained, to a large extent, from studies on actively growing cells and little is known about how cells in the quiescent state repair DNA. Recent findings demonstrated attenuation of DNA repair in quiescent hematopoietic stem cells leading to accumulation of DNA damage, which is repaired upon entry into cell cycle [56]. Furthermore, circulating human B lymphocytes are deficient in nucleotide excision repair (NER) [57], and the ATR-p53 pathway is suppressed in noncycling lymphocytes via ATR downregulation [58]. Mitogen stimulation might also influence the DNA damage response. For instance, phosphorylation of the histone H2AX and ATM activation were strongly amplified during mitogenic stimulation of lymphocytes [59]. Interestingly, microgravity forces growing cells into a condition of metabolic quiescence and strongly affects energy metabolism and DNA repair [17]. Thus, it could be argued that gravity-associated differences in DNA repair pathways can be attributed to the propensity of microgravity to inhibit cell proliferation resulting in an increase in quiescent cells. Since in our experiments we did not (intentionally) stimulate PBMCs to grow, this hypothesis would explain our lack of results supporting gravity-associated changes in gene expression. However, further studies are necessary in order to clarify this observation. 

We found a significant synergy between isoproterenol, microgravity and radiation in their combined effect on *ADBR2* expression. This finding suggests a novel potential player in the regulation of radiation-induced DNA damage response, namely the involvement of β2-adrenergic receptor activation. In particular, we found an increase in ADRB2 mRNA levels after isoproterenol treatment in 1*g*, which was reduced in µg. Upregulation of *ADRB2* gene expression can lead to a higher receptor density and consequently to a higher adrenergic sensitivity. Indeed, acute stimulation of adrenergic receptors induces an increase in receptor density and is predictive of a decrease in lymphocyte proliferation in response to mitogens [60]. Interestingly, we also found an upregulation of *ADRB2* after radiation in 1*g* as well as in µg, although less pronounced in µg. Ligand-independent receptor activation has been observed for some cellular receptors. For example, radiation-induced activation of the endothelial growth factor receptor (EGFR) has been extensively investigated [61,62,63]. However, whether or not radiation increases receptor density and whether or not the effect, if any, is radiation/gravity-dependent, needs to be investigated. Furthermore, it has been shown that chronic stimulation of adrenergic receptors leads to accumulation of DNA strand breaks by MDM2-mediated degradation of p53 [64]. Likewise, our results showed a statistically significant increase in *MDM2* and a decrease *TP53* gene expression after 0.8 and 2 Gy of radiation in both 1*g* and µg conditions. 

The percentage of PBMCs undergoing radiation-induced apoptosis was similar to previously published data [65,66]. Also, in accordance with previous studies [20,25], our results indicate a slight reduction in the percentage of apoptotic cells in simulated microgravity alone, although this effect could not be regarded as statistically significant. Furthermore, as published before [67], we also found that isoproterenol inhibited radiation (2 Gy)-induced apoptosis in cells incubated in 1*g* (Iso × R (1*g*)) although this was not the case in µg. Isoproterenol-mediated inhibition of radiation-induced apoptosis could be due to an increase in cAMP signaling, which has been shown to inhibit apoptosis by reducing ATM-dependent activation of NF-κB [68] and by preventing p53 accumulation [34]. Interestingly, propranolol, a β-adrenergic receptor antagonist, combined with radiation increased apoptosis in human gastric adenocarcinoma cell lines [69], suggesting an antiapoptotic function of β-adrenergic receptor in irradiated cells. However, without radiation isoproterenol induces apoptosis in 1*g* but not in µg leading to a significant interaction between response to isoproterenol and microgravity (Iso × g). It has been shown that stimulation of β-adrenergic receptor induces apoptosis in thymocytes [33] and the catecholamine dopamine and dobutamine induce apoptosis in peripheral blood mononuclear cells after 24 and 48 h of ex vivo incubation [67]. The lack of isoproterenol-mediated inhibition of apoptosis in µg could simply reflect the fact of lower apoptotic cells in microgravity. Our results do not support previous findings reporting an impaired apoptosis response to radiation under simulated microgravity [16,25]. As mentioned above, most simulated microgravity studies have been conducted using proliferative cells, either cell lines or ex vivo stimulated primary lymphocytes. It is well known that stimulation with growth factors can influence cell death [70,71,72]. Furthermore, stimulated lymphocytes go into a p53-dependent, p21-mediated growth arrest, whereas nonstimulated lymphocytes rapidly go into p53-dependent apoptosis [73]. Moreover, radiation-induced apoptosis in lymphocytes can be mediated by p53-dependent or p53-independent mechanisms [74]. We observed a radiation-induced downregulation of p53 in 1*g* as well as in µg, which tended to be more pronounced in microgravity (Figure 3). Interestingly, the ATR-p53 signal pathway is downregulated in quiescent lymphocytes, especially after radiation [58]. Jones et al. suggest a repressed DNA damage response might protect quiescent lymphocytes from the potential induction of p53-dependent apoptosis despite an endurable DNA damage [58] and another study shows activation of antiapoptotic genes in the quiescence state [75]. Interestingly, we found increased amount of DNA strand breaks after 24 h of microgravity exposure suggesting that adaptation to microgravity might be accompanied by accumulation of DNA damage and/or less effective DNA repair mechanisms. No significant synergistic effects were found when the low radiation dose (0.8 Gy) was applied. This could be due to the differences in the responses of different subpopulations to low- and high-dose radiation. In an in vivo mouse study CD8(+) T and B cells were rather resistant to low doses but were very sensitive to 2 Gy, while NK cells, DCs and regulatory T cells (Tregs) cells were much more resistant to high doses [22].

Cytokines are molecules produced in response to stimuli that play a crucial role in regulating cell adhesion, immune recognition, cell death and survival, cell cycle arrest and proliferation, and metabolism [76]. Ionizing radiation stimulates secretion of pro-inflammatory cytokines in a dose-dependent manner [77]. Cytokine production in response to low-dose/low-dose rate (Co γ-rays 0.01 Gy, 0.03 cGy/h), with and without acute 2 Gy proton (1 Gy/min) or γ-ray (0.9 Gy/min) irradiation has been investigated also, concluding that the release of at least some cytokines in response to acute 2 Gy radiation is dependent on the radiation quality at the time of assessment, and on the pre-exposure to low-dose radiation with γ-rays [78]. Isoproterenol suppresses release of cytokines from concanavalin A-activated T cells [79], and microgravity-induced changes in cytokine release have been reported recently in mitogen-stimulated cells [80]. However, radiation-induced cytokine production under microgravity conditions has not been investigated. We found increased concentration of some cytokines after radiation but only in cells incubated in microgravity, which was completely abolished by previous treatment with isoproterenol. These findings support and complement in vivo data obtained from mouse hind limb suspension microgravity-models. The combination of 2 Gy (but not low dose) of gamma or proton radiation and hind limb unloading led to an increase in circulating TNFα, while when used separately did not show any effect [81]. Corresponding to protein levels, we also found a radiation-induced increase in *TNF* gene expression only in microgravity (data not shown). 

**Figure 5:**
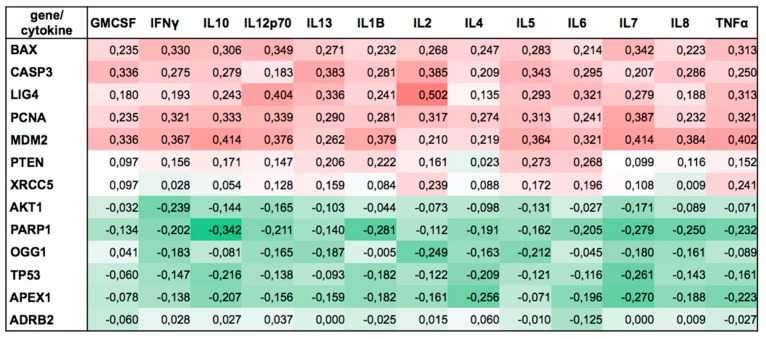
Association between changes in cytokines and corresponding changes in gene expression over the eight experimental conditions for the group of samples irradiated with 2 Gy. BAX, CASP3, PCNA, LIG4, and MDM2 gene expressions were positively associated, while AKT1, TP53, PARP1, OGG1, and APXE1 were negatively associated with cytokines.

However, cytokine levels relate to cell functionality. There is growing evidence linking DNA damage response elements with inflammatory responses. TGF-β, IL-6, and thrombopoietin influence ATM-dependent DNA damage response, and have been proposed as biomarkers of radioresistance [82]. Furthermore, NER, responsible for the removal of UV-mediated DNA damage, can be modulated by cytokines, including IL-12, IL-18, and α-melanocyte-stimulating hormone [83]. In addition, IL-1α can act as an intracellular DNA-damage sensor, signaling cellular genotoxic stress [84]. A link between the immune system and DNA repair can be found in the V(D)J rearrangement process, responsible for the production of a large repertoire of antigen receptors with different specificities, a requirement for the normal functioning of the immune system. DNA repair proteins such as DNA-PKcs, Ku70, Ku80, XRCC4, LIG4, and Artemis are also involved in the V(D)J recombination [85]. Here, we found associations between intrasubject changes in cytokines and corresponding changes in genes over the 8 experimental conditions. Expressions of *BAX*, *CASP3*, *PCNA*, *LIG4*, and *MDM2* were positively correlated with all 13 cytokines studied. Conversely, expressions of *AKT1*, *TP53*, *PARP1*, *OGG1*, and *APXE1* were negatively correlated with these cytokines (Figure 5). Interestingly, for each gene, the sign of the correlation was consistent among all analyzed cytokines. Although it was not the intent of this study to investigate relations between cytokines and DNA damage response, our findings suggest a new link between immune cells and DNA damage response under microgravity conditions. Therefore, we point this out as an interesting starting point for future research necessary for understanding this relationship. 

In summary, our results indicate synergistic effects between microgravity, radiation and adrenergic receptor activation. However, our study has some limitations. All measurements were conducted on samples of freshly isolated PBMCs from different subjects. Within dose groups, linear mixed model regression analyses take baseline interindividual differences into account; therefore uncertainties in the estimates of the contrasting effects are due to random interactions between the individuals and the experimental conditions. 

The widely recommended housekeeping genes (HKG) *ACTB* and *GAPDH*, especially *ACTB*, turned out to be too dependent on the experimental conditions to provide reliable normalization of expression within PCR plates. Instead, we chose HKG empirically to be *ATM*, *CREB1*, and *PRKACA* using the dual criteria of relatively low dependence on the experimental conditions, while still being sensitive to overall differences between PCR plates. Choosing HKG empirically is a strategy that is gaining acceptance among a wide variety of modern PCR studies. However, given the small number of genes analyzed here, our results need to be confirmed. Nevertheless, the fact that our data confirmed radiation-induced dysregulation of genes involved in DNA damage response in a dose dependent manner supports our strategy as a plausible alternative way for evaluating gene expression analysis. 

Of note, our study was exploratory, being designed to identify key elements of the DNA damage response that appeared to be significantly affected by one or more of the mentioned factors. Because of testing multiplicity, even with moderate control of the false-discovery rate, verification of these results through additional controlled studies is recommended.

## 4. Materials and Methods

### 4.1. Isolation of PBMCs from Whole Blood

Blood was obtained from volunteers in accordance with accepted ethical and humane practices. Ethical approval was obtained from NASA Johnson Space Center Institutional Review Board, protocol number Pro0614. Whole blood samples were drawn into BD Vacutainer^®^ or CPT™ Mononuclear Cell Preparation Tubes containing sodium heparin (BD Biosciences, Franklin Lakes, NJ, USA). Peripheral blood mononuclear cells (PBMC) were isolated following the manufacturer’s instructions by directly centrifuging the CPT™ to obtain a density gradient or, for the blood collected in the BD Vacutainer^®^ tubes, using the Ficoll-Paque^TM^ PLUS (GE Healthcare, Uppsala, Sweden). Isolated PBMCs were transferred into 15-mL tubes and washed with phosphate buffered saline (PBS) (Gibco^®^, Waltham, MA, USA). Tubes were then centrifuged at 300× *g* for 10 min, the supernatant was removed, and the cell pellet was resuspended in 40 mL of TexMacs medium (Miltenyl Biotec, Auburn, CA, USA). Cells were counted using Guava ViaCount technology (EMD Millipore Co., Hayward, CA, USA). For DNA strand breaks analysis, isolated cells were suspended in 1 mL of freezing medium containing 20% Roswell Park Memorial Institute medium (RPMI-1640) medium, 10% dimethyl sulfoxide (DMSO), and 70% fetal calf serum (FCS), and stored overnight at −80 °C in a Mr. Frosty™ Freezing Container (Thermo Fisher Scientific, USA). The cells were then transferred to a liquid nitrogen tank at −180 °C until shipment to Konstanz (Germany), where cells were kept at −180 °C until analysis. Then, PBMCs were carefully thawed by immersing cryovials in a water bath at 37 °C until a small amount of ice remained in the cryovial and thereafter adding 1 mL of thawing medium (90% RPMI and 10% FCS). After 1 min the cell suspension was transferred into a polypropylene 15 mL tube and thawing medium was added stepwise (1 mL, 1 min later additional 2 mL, 1 min later additional 4 mL were added). The tube was centrifuged at 300× *g* for 10 min. The cell pellet was gently resuspended in 1 mL RPMI medium, and the cell concentration and viability (determined by electric current exclusion) were assessed using CASY cell counter technology (Innovatis, Switzerland). No significant difference was detected in the cell vitality of control cells after thawing (68.2 ± 7% and 65.2 ± 5.6% in simulated microgravity and 1*g*, respectively).

### 4.2. Experimental Design

**Figure 6:**
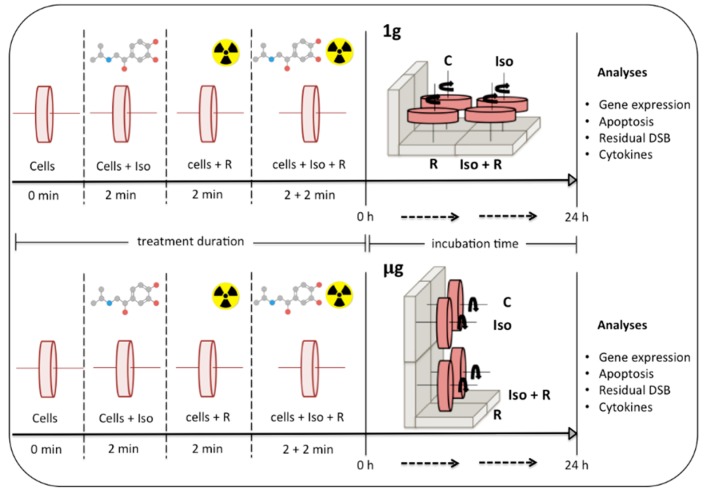
Schematic representation of the treatment conditions. Cells were distributed in eight RCCSVs. Isoproterenol treatment (Iso) and radiation exposure (R) were performed separately and in combination (Iso + R). For the combined treatment, cells were irradiated for 2 min immediately after 2 min isoproterenol (Iso) treatment. Vessels were placed in an incubator and rotated at 8.5 rpm on a vertical (Earth gravity = 1*g*) or on a horizontal axis (simulated microgravity = µg). Vessels with nontreated cells (C) were incubated in 1*g* and in µg. After 24 h cells were recovered from the vessels and analyses were performed (DSB = DNA strand breaks).

To mimic the absence of gravity, cell suspensions were added to rotating cell culture system vessels (RCCSVs) (Synthecon Inc., Houston, TX, USA) similar to the RWVs originally developed by NASA. Four vessels were allocated to Earth gravity experimental conditions (1*g*) and were rotated horizontally, while four others were allocated to simulated microgravity conditions (µg) and were rotated vertically. For each level of gravity (1*g* and µg), cells in vessels were either, (1) not treated (control), (2) treated with 10µM (-)-isoproterenol hydrochloride (Sigma-Aldrich, Milwaukee, WI, USA), (3) irradiated (0.8 or 2 Gy), or (4) treated with 10µM (-)-isoproterenol hydrochloride and immediately irradiated (0.8 or 2 Gy) (Figure 6). Vessels were placed on the rotary cell culture systems (Synthecon Inc., Houston, TX, USA). After treatment and/or radiation all vessels rotated synchronously at a speed of 8.5 rpm for 24 h in an incubator (37 °C, 5% CO_2_, and 95% relative humidity). After incubation, cells were recovered from the RCCSVs and cell concentration and viability was determined using Guava ViaCount technology (EMD Millipore, Hayward, CA, USA) prior to further analyses.

### 4.3. Gene Expression

After incubation, cells were centrifuged at 300× *g* for 10 min after which the supernatant fluid was carefully removed. RNA was isolated from each cell pellet using AllPrep DNA/RNA/miRNA Universal Kit (Quiagen, Hilden, Germany). RNA concentration was measured using a NanoDrop Lite Spectrophotometer (Thermo Fisher Scientific, Waltham, MA, USA). Reverse transcription was performed using the miScript II RT Kit (Quiagen, Hilden, Germany), and conversion to cDNA was performed in the DNA Engine^®^ Thermal Cycler (BioRad, Hercules, CA, USA). cDNA was diluted in 40 µL RNase-free water. The PCR reagent consisted of 12.5 µL 2xQuantiTect SYBR Green PCR Master Mix, 7.5 µL RNase-free water, 2.5 µL 10x Primer Assay, and 2.5 µL Template cDNA. PCR was performed in a CFX96TM Thermal Cycler (BioRad, Hercules, CA, USA). Genes of interest were selected according to the following criteria: (i) genes that are major players and representative of the pathways described in Secs 1.1 and 1.2); (ii) genes that are known to be involved in two or more of these relevant suggested pathways; (iii) genes that are expected to undergo adaptive responses; (iv) genes coding a considerable number of proteins known to be regulated at the mRNA level.

### 4.4. Apoptosis

The percentage of dead cells after incubation was determined by diluting samples 1:1 with Guava Nexin^®^ reagent (EMD Millipore Co., Billerica, MA, USA). These mixtures were incubated for twenty minutes. Apoptotic cells were counted in Guava-PCA machine (EMD Millipore, Hayward, CA, USA). 

### 4.5. Detection of DNA Strand Breaks

DNA strand breaks were detected using the automated version of the “Fluorimetric detection of Alkaline DNA Unwinding” (FADU) assay [86,87]. This assay is based on controlled DNA unwinding that starts at DNA strand breaks. SybrGreen^®^ (MoBiTec, Göttingen, Germany) was used as the marker for double stranded DNA. A decrease in the fluorescence intensity indicates an increase in DNA unwinding and consequently a greater number of strand breaks. The fluorescence signal was transformed into equivalent radiation dose units (Gy) [88]. 

### 4.6. Cytokine Quantification

Medium supernatant was used for cytokine measurements. The concentrations for 13 cytokines were determined simultaneously in a 96-well plate in duplicate using a commercially available multiplex bead immunoassay (R&D Systems). Briefly, 50 µL of medium supernatant were incubated with 13 sets of beads each precoated with specific antibodies against cytokines of interest. The bead sets fluoresce at different wavelengths so that individual cytokines can be identified. After a washing step, the bead–cytokine complex was incubated with fluorescence secondary antibodies specific for each cytokine that fluoresce along a single channel distinct from the bead populations. Fluorescence, indicating relative concentration, was assessed using a Luminex 100 instrument (Luminex, Inc. Austin, TX, USA). Cytokines were selected based on their different types of biological responses including innate immunity, adaptive immunity, and growth factors, and based on their physiological functions: pro- versus anti-inflammatory properties.

### 4.7. Statistical Analysis

Each blood sample was split into eight subsamples following a repeated-measures 2^3^-factorial design with radiation (yes, no), isoproterenol (yes, no), and simulated microgravity (yes, no) as factors (Figure 6). For all analyses (gene expression, apoptosis, cytokines, and DNA strand breaks), mixed model regression with bootstrapped standard errors (200 reps) was used to make inference on contrasts involving the experimental factors. Mixed model random effects were modeled at the sample level (apoptosis, cytokines, strand breaks) and at the PCR-plate level (gene expression). To account for test multiplicity, we used either the method of Benjamini Y., A. Krieger, and D. Yekutieli [89] to control the false-discovery rate (FDR) or the method of Holm [90] to control the family-wise Type I error rate (FWER) depending on the number of tests run. A summary of statistical methods applied for each cellular parameter is indicated in Table 6. The nomenclature for experimental factors and analyzed contrasts is elucidated in Table 7. 

### 4.8. Comparison of Cytokine and Gene Expression Data

In addition to the separate analyses for each cellular parameter, we also used the Somers D [91,92] statistic to create the table of associations (Figure 5) between within-subject changes in cytokines and corresponding changes in genes over the eight experimental conditions. 

## 5. Conclusions

Psychological stress provokes the release of neuroendocrine hormones, such as catecholamines activating adrenergic pathways, a process that is fundamental for the initiation of the “fight-or-flight” response. This adrenergic activation is not only the main mechanism in the initiation and execution of the flight-or-fight response but it also plays an important role in immune regulation [93,94,95], bone homeostasis [96,97,98], muscle hypertrophy and atrophy [99,100], the cardiovascular system [101], neuronal activity in primary motor cortex [102], and neuronal plasticity and memory [103,104,105] among others. Here we report the synergistic effects of radiation, microgravity and adrenergic receptor stimulation on DNA damage response in quiescent human primary lymphocytes. Synergistic effects of activation of adrenergic receptor and DNA damage response can be relevant in cancer patients undergoing chemo- and/or radiotherapy. An indication for this statement is the observation that the use of -blockers is associated with improved metastasis-free, disease-free, and overall survival in 722 patients with non-small-cell lung cancer who received definitive radiotherapy [106]. This is also supported by a mouse study showing that propranolol, a β_2_-AR antagonist, potentiates the antitumor effects of radiotherapy in gastric cancer by inhibiting NF-κB expression and its downstream genes: VEGF, EGFR, and COX-2 [107]. Regarding microgravity and cancer, numerous studies report a microgravity-induced cell growth inhibition in cancer cells [108]. Therefore, implementation of RWV in ex vivo and in vitro models can help in understanding not only the effect of microgravity but also the cellular mechanisms behind diseases such as cancer and thus, developing new therapeutic concepts. 

RWVs also provide a realistic model that reproduces some of the effects observed in “real” space microgravity including leukocyte proliferation, cytokine production, and leukocyte subset distribution [109]. Therefore our results are also relevant for the field of space medicine. Understanding the effect of isoproterenol on radiation-induced DNA damage response and T-cell activation in microgravity is essential for developing countermeasures and needs to be investigated further. For instance, since stimulation of the β-adrenergic receptor is followed by an increase in cAMP, drugs based on the manipulation of cAMP/PKA activity could be an option for space medicine. Interestingly, cAMP/PKA pathway activation in human mesenchymal stem cells in vitro results in robust bone formation in vivo [110]. 

## Figures and Tables

**Table 1:** Estimated change (diff) in mean transformed apoptosis rate and DNA strand breaks. Effects listed below are described in Table 7.

Effect	Diff	*p* Value
**Apoptosis 0.8 Gy**		
R (1*g*)	0.13	**0.0000138 #**
µg	−0.1	0.0156
Iso × *g*	−0.1	0.0202
R (µg)	0.13	0.0498
Iso (1*g*)	0.06	0.103
Iso (µg)	−0.05	0.149
Iso × R × *g*	0.06	0.278
Iso (1*g*, R)	0.04	0.313
Iso × R (µg)	0.04	0.34
Iso × R (1*g*)	−0.02	0.684
Iso (µg, R)	−0.01	0.724
R × *g*	0	0.941
**Apoptosis 2 Gy**		
R (µg)	0.37	**5.32 × 10^−15^ #**
R (1*g*)	0.39	**0.0000189 #**
Iso × R (1*g*)	−0.2	**0.0000635 #**
Iso (1*g*)	0.11	**0.000782 #**
Iso × *g*	−0.12	**0.00092 #**
Iso (1*g*, R)	−0.09	**0.00276 #**
Iso (µg, R)	−0.06	0.00955
Iso × R × *g*	0.15	0.0148
µg	−0.07	0.0156
Iso × R (µg)	−0.05	0.123
Iso (µg)	−0.01	0.218
R × *g*	−0.02	0.612
**DNA strand breaks 2 Gy**		
µg	0.78	**0.000 #**
Iso (1*g*, R)	0.50	**0.000 #**
Iso (µg)	−0.60	**0.000 #**
Iso × R (µg)	−0.37	**0.007 #**
R (1*g*)	0.35	**0.010 #**
R (µg)	0.30	0.024
Iso (1*g*)	−0.18	0.167
Iso × R (1*g*)	0.15	0.259
Iso (µg, R)	−0.07	0.607

With control of the FWER (family-wise error rate—see statistical methods) to 5% (Holm) significant changes are highlighted with #. For apoptosis rate: n = 15 independent experiments for 0.8, n = 16 independent experiments for 2 Gy. *p*-value threshold: 0.0045 for 0.8 Gy and 0.0083 for 2 Gy. For DNA strand breaks: n = 6 independent experiments for 2 Gy. *p*-value threshold: 0.0125.

**Table 2:** Significant differences in mean transformed gene expression (0.8 Gy). Effects listed below are described in Table 7.

Gene	Effect	Diff	Se	Df	*p* Value
*ADRB2*	R (µg)	0.3327	0.0715	36.7	0.0000417
*ADRB2*	Iso (1 g)	0.3107	0.0781	58.4	0.000195
*BAX*	R (µg)	1.4426	0.1567	9.69	4.22 × 10^−6^
*BAX*	R (1*g*)	1.2942	0.1659	6.76	0.000128
*CASP3*	Iso (µg)	0.3497	0.094	20.5	0.0013
*CASP3*	R (µg)	0.435	0.1185	15.2	0.00224
*CASP3*	Iso × R (µg)	−0.4005	0.1342	61.3	0.00408
*LIG4*	R × *g*	0.2239	0.0713	58.6	0.00264
*LIG4*	Iso (1*g*, R)	−0.3771	0.0976	10.2	0.00302
*LIG4*	Iso × R (1*g*)	−0.308	0.0989	16.3	0.00655
*LIG4*	Iso (µg, R)	−0.3202	0.1	12.5	0.00722
*MDM2*	R (1*g*)	0.8083	0.0571	67.4	7.40 × 10^−22^
*MDM2*	R (µg)	0.8957	0.1039	12.9	1.01 × 10^−6^
*MDM2*	Iso (1*g*)	−0.1626	0.0553	16.3	0.00938
*OGG1*	Iso (1*g*)	−0.22	0.0722	49.6	0.00368
*PARP1*	R (1*g*)	−0.1836	0.0468	44.9	0.000295
*PARP1*	µg	−0.2918	0.0809	21.6	0.0016
*PARP1*	Iso (1*g*, R)	−0.129	0.0427	64.3	0.00365
*PARP1*	Iso (µg, R)	−0.1946	0.0647	25.4	0.00584
*PARP1*	Iso (1*g*)	−0.1066	0.0409	91.6	0.0107
*PCNA*	R (1*g*)	0.6586	0.0666	55.3	7.80 × 10^−14^
*PCNA*	R (µg)	0.8327	0.1228	9.58	0.00006
*PTEN*	Iso (1*g*, R)	−0.1878	0.0446	18.5	0.000494
*PTEN*	Iso × R (µg)	−0.3164	0.0717	9.44	0.00151
*PTEN*	Iso × *g*	0.2721	0.0893	46.3	0.0038
*PTEN*	Iso (µg)	0.179	0.059	35.4	0.00453
*TP53*	R (1*g*)	−0.349	0.0851	22.2	0.000466
*XRCC5*	Iso (1*g*, R)	−0.3624	0.0402	26.2	1.62 × 10^−9^
*XRCC5*	Iso × R (µg)	−02794	0.049	30.1	3.18 × 10^−6^
*XRCC5*	Iso (µg, R)	−0.2963	0.0526	16.6	0.0000326
*XRCC5*	Iso × R (1*g*)	−0.3697	0.0794	23.2	0.000108

Controlling the false-discovery rate (FDR) to 5% corresponded to a *p*-value threshold of 0.0113, which in turn led to the identification of 31 significant contrasts among the 180 that were estimated. n = 9 independent experiments. Iso = isoproterenol, R = 0.8 Gy radiation, *g* = gravity. *p*-value threshold *p* < 0.0113 after controlling the FDR to 5%. diff = estimate difference, se = standard error, df = degrees of freedom.

**Table 3:** Significant differences in mean transformed gene expression (2 Gy). Effects listed below are described in Table 7.

Gene	Effect	Diff	Se	Df	*p* Value
*ADRB2*	R × *g*	−0.3369	0.0992	34.4	0.00174
*ADRB2*	R (µg)	0.481	0.0848	34.4	2.20 × 10^−6^
*ADRB2*	Iso (1*g*)	0.5047	0.1009	33.7	0.0000175
*ADRB2*	Iso (µg, R)	0.5138	0.1528	19.1	0.00324
*ADRB2*	Iso × R × *g*	0.6219	0.2297	48.1	0.00935
*ADRB2*	R (1*g*)	0.8179	0.1239	19.2	2.42 × 10^−6^
*AKT1*	R (1*g*)	−0.2202	0.0598	18.9	0.00158
*AKT1*	R (µg)	−0.1696	0.0509	29.3	0.00234
*APEX1*	R (µg)	−0.4306	0.054	52.7	1.27 × 10^−10^
*BAX*	R (1*g*)	1.9229	0.102	5.96	1.54 × 10^−6^
*BAX*	R (µg)	1.9254	0.0481	28.2	1.97 × 10^−26^
*CASP3*	Iso (µg, R)	−0.4001	0.0794	55.4	5.38 × 10^−6^
*MDM2*	R (µg)	1.6218	0.0694	9.65	8.14 × 10^−10^
*MDM2*	R (1*g*)	1.6469	0.1196	7.75	1.00 × 10^−6^
*PARP1*	R (µg)	−0.3947	0.0465	32.5	9.41 × 10^−10^
*PARP1*	R (1*g*)	−0.2701	0.0602	20.8	0.000208
*PCNA*	R (1*g*)	1.2425	0.0831	13.2	1.19 × 10^−9^
*PCNA*	R (µg)	1.3845	0.046	80	0
*TP53*	R (µg)	−0.6312	0.056	47.6	5.04 × 10^−15^
*TP53*	R (1*g*)	−0.4132	0.0749	25.7	8.95 × 10^−6^
*XRCC5*	Iso (µg, R)	−0.2727	0.0225	146	7.45 × 10^−24^
*XRCC5*	Iso × R (µg)	−0.2678	0.0538	30.4	0.000024

Controlling the false-discovery rate (FDR) to 5% corresponded to a *p*-value threshold of 0.0098, which in turn led to the identification of 22 significant contrasts among the 180 that were estimated. n = 10 independent experiments. Iso = isoproterenol, R = 2 Gy radiation, *g* = gravity. *p*-value threshold *p* < 0.0098 after controlling the FDR to 5%. Diff = estimated difference, se = standard error, df = degrees of freedom.

**Table 4:** Significant differences in mean transformed cytokine concentration (0.8 Gy). Effects listed below are described in Table 7.

Cyt	Effect	Diff	Se	Df	*p* Value
GM-CSF	R (µg)	−0.3165	0.0939	15.5	0.00404
IL-10	R (µg)	0.9723	0.3034	23	0.00393
IL-12p70	Iso (µg, R)	−2	0.3567	30.7	3.87 × 10^-6^
IL-12p70	Iso (µg)	−2.6	0.4945	108	7.38 × 10^-7^
IL-12p70	R (µg)	2.3	0.5767	32.6	0.000354
IL-1B	R (µg)	1.309	0.3461	8.67	0.00463
IL-1B	R × *g*	1.4022	0.4936	117	0.00531
IL-2	R (µg)	0.1426	0.0432	65.4	0.00156
IL-4	R (µg)	0.0851	0.0262	42.4	0.00223
IL-5	Iso (µg)	0.1321	0.0407	105	0.00155
IL-6	Iso (µg, R)	−2	0.4747	11.2	0.0014
IL-6	Iso (µg)	−2	0.5868	26.8	0.00208
IL-6	R (µg)	2	0.4385	11.8	0.000685
TNFα	µg	−0.0355	0.0098	26.1	0.00118
TNFα	Iso (µg, R)	0.0359	0.007	97.8	1.41 × 10^-6^
TNFα	Iso (µg)	0.0402	0.0047	542	7.43 × 10^-17^
TNFα	Iso (1*g*, R)	0.0675	0.0189	14.7	0.0029
TNFα	R (µg)	−0.028	0.0069	13.3	0.0013

Controlling the false-discovery rate (FDR) to 5% corresponded to a *p*-value threshold of 0.0068, which in turn led to the identification of 18 significant contrasts among the 144 that were estimated. n = 10 independent experiments. cyt = cytokine, Iso=isoproterenol, R = 0.8 Gy radiation, *g* = gravity, *p*-value threshold *p* < 0.0068 after controlling the FDR to 5%. diff = estimated difference, se = standard error, df = degrees of freedom.

**Table 5:** Significant differences in mean transformed cytokine concentration (2 Gy). Effects listed below are described in Table 7.

Cyt	Effect	Diff	Se	Df	*p* Value
GM-CSF	Iso (µg, R)	−4.4	0.5784	31.2	1.36 × 10^−8^
GM-CSF	Iso (1*g*)	−2.8	0.624	24.5	0.000147
GM-CSF	Iso × R (µg)	−2.95	0.8635	43.9	0.00138
GM-CSF	Iso × R × *g*	−6.5	1.5547	32.9	0.000202
GM-CSF	R (µg)	2.85	0.4337	16.1	6.28 × 10^−6^
GM-CSF	R × *g*	4.15	0.9974	18.5	0.000556
IL-10	Iso (µg, R)	−3.05	0.6062	7.7	0.00114
IL-10	Iso (µg)	−1.5	0.4324	36.2	0.00137
IL-10	R (µg)	2.9	0.3257	19.9	2.26 × 10^−8^
IL-12p70	Iso (µg, R)	0.2468	0.0496	8.39	0.000941
IL-12p70	Iso × R (µg)	0.171	0.049	15.4	0.0032
IL-12p70	R (µg)	−0.2024	0.0416	18.8	0.000111
IL-13	Iso (µg, R)	−3.55	0.4825	90.5	8.18 × 10^−11^
IL-13	Iso (1*g*)	−2.8	0.5063	68.6	5.42 × 10^−7^
IL-1B	Iso (µg, R)	−2.4713	0.5444	13.6	0.000493
IL-1B	Iso (1*g*)	−0.9541	0.214	73.4	0.0000292
IL-1B	Iso × R (µg)	−2.1307	0.5551	12.8	0.0021
IL-1B	R (µg)	2.709	0.4346	11.7	0.0000485
IL-2	µg	−0.1161	0.0403	106	0.00475
IL-2	Iso (µg. R)	−0.2931	0.0611	20.6	0.000103
IL-2	Iso (1*g*)	−0.1642	0.0402	48	0.000164
IL-2	R (µg)	0.2543	0.0444	51.2	5.46 × 10^−7^
IL-2	R × *g*	0.3121	0.0776	22.8	0.000542
IL-4	Iso (µg, R)	−0.1665	0.0475	12	0.00432
IL-4	Iso (µg)	−0.0993	0.0326	29.2	0.00488
IL-4	R (µg)	0.1426	0.0414	44.9	0.00124
IL-5	Iso (µg, R)	−4.4	0.5475	29.3	6.85 × 10^−9^
IL-5	Iso (1*g*)	−3.1	0.7242	29.5	0.000181
IL-5	Iso × R × *g*	−5.9	1.5538	23	0.000932
IL-5	R (µg)	2.4	0.5134	60.8	0.0000168
IL-6	Iso (µg, R)	−3.7	0.7387	8.3	0.000933
IL-6	Iso (µg)	−2.3	0.3979	240	2.30 × 10^−8^
IL-6	Iso (1*g*)	−3.4	0.587	11.1	0.000115
IL-6	R (µg)	2.3	0.3138	41.5	5.26 × 10^−9^
IL-7	µg	0.0751	0.0154	73.1	6.50 × 10^−6^
IL-7	Iso (µg, R)	0.1521	0.0246	23.2	2.47 × 10^−6^
IL-7	Iso (1*g*)	0.1062	0.0216	23.3	0.0000547
IL-7	Iso × *g*	−0.0889	0.023	30.5	0.000532
IL-7	Iso × R (µg)	0.1348	0.0289	23.2	0.000108
IL-7	Iso × R × *g*	0.2149	0.0417	20.5	0.0000453
IL-7	R (µg)	−0.161	0.0216	30.3	2.47 × 10^−8^
IL-7	R × *g*	−0.1724	0.0254	36.2	6.06 × 10^−8^
IL-8	Iso (µg, R)	−3.2	0.5387	21.8	5.82 × 10^−6^
IL-8	R (µg)	2.5	0.6377	53.7	0.000252
TNFα	Iso (µg, R)	−4	0.4773	16.4	2.48 × 10^−7^
TNFα	Iso (µg)	−2.9	0.511	26.6	5.24 × 10^−6^
TNFα	Iso (1*g*)	−4.2	0.5913	24.1	2.38 × 10^−7^
TNFα	R (µg)	2.7	0.4469	22.6	3.96 × 10^−6^
TNFα	R × *g*	2.8	0.693	10.2	0.00227

Controlling the false-discovery rate (FDR) to 1% corresponded to a *p*-value threshold of 0.0049, which in turn led to the identification of 49 significant contrasts among the 144 that were estimated. n = 10 independent experiments. cyt = cytokine, Iso = isoproterenol, R = 2 Gy radiation, *g* = gravity, *p*-value threshold *p* < 0.0049 after controlling the FDR to 1%. diff = estimated difference, se = standard error, df = degrees of freedom.

**Table 6:** List of statistical methods applied for each measured parameter.

Cellular Parameters	Nr. of Experiments Performed		Statistical Model	Dependent Variable	Multiple Testing
	0.8 Gy	2 Gy			
Apoptosis and DNA strand breaks	15	16	Mixed model regression. Random effects at sample level	2 sin^−1^ √p where p is the proportion of cells undergoing apoptosis	FWER control to 5% (12 tests)
	--	6	Mixed model regression. Random effects at sample level.	equivalent dose (Gy)	FWER control to 5% (9 tests)
Gene expression	9	10	Mixed model regression. Random effects at PCR-plate level.	log_2_ cycles normalized to average values of three housekeeping genes	FDR control to 5% (180 tests)
Cytokines	10	10	Mixed model regression. Random effects at sample level.	log concentration	FDR control to 5% (0.8 Gy) control to 1% (2 Gy) (144 tests)

Statistical significance for differences in apoptosis, DNA strand breaks, gene expression, and cytokines was calculated applying mixed model regression analyses.

**Table 7:** Nomenclature for experimental factors and analyzed effects.

Analysis Effects (Contrasts)	Description
R (µg)	Effect of radiation alone in simulated microgravity
R (1*g*)	Effect of radiation alone in Earth gravity
Iso (µg)	Effect of isoproterenol alone in simulated microgravity
Iso (1*g*)	Effect of isoproterenol alone in Earth gravity
Iso (µg, R)	Effect of isoproterenol on irradiated cells in simulated microgravity
Iso (1*g*, R)	Effect of isoproterenol on irradiated cells in Earth gravity
Iso × R (µg)	Synergistic effect of Isoproterenol and radiation in simulated microgravity
Iso × R (1*g*)	Synergistic effect of Isoproterenol and radiation in Earth gravity
µg	Average effect of microgravity over all combinations of the other factors (R and Iso)
Iso × *g*	Average synergistic effect of Isoproterenol and gravity for non-irradiated cells
R × *g*	Average synergistic effect of radiation and gravity for cells without Isoproterenol
Iso × R × *g*	Three-way synergistic effect of Isoproterenol, radiation, and gravity

The 12 contrasts represent difference of population means and are estimated based on the mean of each experimental condition C, Iso, R, and R + Iso.
